# Pan-European Distribution of White-Nose Syndrome Fungus (*Geomyces destructans*) Not Associated with Mass Mortality

**DOI:** 10.1371/journal.pone.0019167

**Published:** 2011-04-27

**Authors:** Sébastien J. Puechmaille, Gudrun Wibbelt, Vanessa Korn, Hubert Fuller, Frédéric Forget, Kristin Mühldorfer, Andreas Kurth, Wieslaw Bogdanowicz, Christophe Borel, Thijs Bosch, Thomas Cherezy, Mikhail Drebet, Tamás Görföl, Anne-Jifke Haarsma, Frank Herhaus, Guénael Hallart, Matthias Hammer, Christian Jungmann, Yann Le Bris, Lauri Lutsar, Matti Masing, Bart Mulkens, Karsten Passior, Martin Starrach, Andrzej Wojtaszewski, Ulrich Zöphel, Emma C. Teeling

**Affiliations:** 1 School of Biology and Environmental Science, University College Dublin, Dublin, Ireland; 2 Conway Institute of Biomolecular and Biomedical Research, Dublin, Ireland; 3 Leibniz Institute for Zoo and Wildlife Research, Berlin, Germany; 4 Office for Faunistic and Landscape Ecology, Schöneberg, Germany; 5 Plecotus Working Group, Association Natagora, Brussels, Belgium; 6 Robert Koch Institute, Berlin, Germany; 7 Museum and Institute of Zoology, Polish Academy of Sciences, Warszawa, Poland; 8 Commission de Protection des Eaux, du Patrimoine, de l'Environnement, du Sous-sol et des Chiroptères – Lorraine, Velaine-en-Haye, France; 9 Dutch Bat Workers Group, Nijmegen, The Netherlands; 10 Coordination Mammalogique du Nord de la France, Béthune, France; 11 Podilski Tovtry National Nature Park, Kamenets-Podilsky, Ukraine; 12 Nature Conservation Foundation of Tolna County, Szekszárd, Hungary; 13 Centre for Ecosystem Studies, Alterra and Wageningen University, Wageningen, The Netherlands; 14 Biology Station Oberberg, Nümbrecht, Germany; 15 Société d'Etude et de Protection de la Nature en Thiérache, Le Chaudron, Origny-en-Thiérache, France; 16 Department of Biology, Center for Bat Conservation in Northern Bavaria, Erlangen University, Erlangen, Germany; 17 Nature and Biodiversity Conservation Union Rhineland-Palatine, Birkenfeld, Germany; 18 Bretagne Vivante SEPNB, Roussimel, Glénac, France; 19 Estonian Fund for Nature, Tartu, Estonia; 20 Sicista Development Centre, Tartu, Estonia; 21 Bat Working Group, Natuurpunt VZW, Belgium; 22 Nature and Biodiversity Conservation Union Southern Lower-Saxony, Nordstemmen, Germany; 23 Biotope Mapping Cooperation, Herford, Germany; 24 Institute of Natural Fibres and Medicinal Plants, Poznan, Poland; 25 Saxonian State Office for Environment Agriculture and Geology, Dresden-Pillnitz, Germany; University of Bern, Switzerland

## Abstract

**Background:**

The dramatic mass mortalities amongst hibernating bats in Northeastern America caused by “white nose-syndrome” (WNS) continue to threaten populations of different bat species. The cold-loving fungus, *Geomyces destructans*, is the most likely causative agent leading to extensive destruction of the skin, particularly the wing membranes. Recent investigations in Europe confirmed the presence of the fungus *G. destructans* without associated mass mortality in hibernating bats in six countries but its distribution remains poorly known.

**Methodology/Principal Findings:**

We collected data on the presence of bats with white fungal growth in 12 countries in Europe between 2003 and 2010 and conducted morphological and genetic analysis to confirm the identity of the fungus as *Geomyces destructans*. Our results demonstrate the presence of the fungus in eight countries spanning over 2000 km from West to East and provide compelling photographic evidence for its presence in another four countries including Romania, and Turkey. Furthermore, matching prevalence data of a hibernaculum monitored over two consecutive years with data from across Europe show that the temporal occurrence of the fungus, which first becomes visible around February, peaks in March but can still be seen in some torpid bats in May or June, is strikingly similar throughout Europe. Finally, we isolated and cultured *G. destructans* from a cave wall adjacent to a bat with fungal growth.

**Conclusions/Significance:**

*G. destructans* is widely found over large areas of the European continent without associated mass mortalities in bats, suggesting that the fungus is native to Europe. The characterisation of the temporal variation in *G. destructans* growth on bats provides reference data for studying the spatio-temporal dynamic of the fungus. Finally, the presence of *G. destructans* spores on cave walls suggests that hibernacula could act as passive vectors and/or reservoirs for *G. destructans* and therefore, might play an important role in the transmission process.

## Introduction

White nose-syndrome (WNS) is a devastating disease causing mass mortalities in hibernating bats in North-America. In May 2009, it was estimated that over one million bats had died from the disease which was first documented in February 2006 at Howe's Cave, West of Albany, New York [1]. A visually conspicuous white fungus grows on the face, ears, or wings of stricken bats with hyphae penetrating deep into the connective tissue of glabrous skin and snout [2] and causing severe damage [3]. The fungus associated with WNS is a newly described, psychrophilic (cold-loving) species (*Geomyces destructans*) [4], closely related to other psychrophilic species of *Geomyces* [5], [6]. Although it is not yet conclusively proven whether *G. destructans* is the causative agent of the disease or if other co-factors are necessary for disease to occur, the fungus is always found on bats at WNS sites where hibernating bats experience mass mortalities [7]. To date, bacteriological, virological, parasitological and pathological evaluations as well as studies of toxic contaminants have not identified the consistent presence of any other agents/cause of death. The lack of evidence for the involvement of other agents or compounds reinforces the suspicion that *G. destructans* is the causative agent of WNS mortality [2], [7], [8], [9].

*Geomyces destructans* has been found in nine species of bats in North-America, from the provinces of Ontario and Quebec in Canada south and west to the states of Tennessee and Oklahoma in the USA [10]. Three recent studies investigating samples collected in 2008–2010 have shown that *G. destructans* was also present in six European countries (France, Germany, Switzerland, Czech Republic, Slovakia & Hungary) [6], [11], [12]. Nevertheless, the geographic coverage of these studies was limited and the extent of the distribution of *G. destructans* in Europe remains poorly known. In this paper, we combine previously published data on the distribution of *G. destructans* in Europe [6], [11], [12] with new data from twelve countries covering 2,400 km from West to East (France to Turkey) and 1,900 km from North to South (Estonia to Turkey) to demonstrate the widespread presence of *G. destructans* on multiple species of hibernating bats in Europe without associated mass mortality.

## Results

### Review of data on *Geomyces destructans* in European bats, 2008–2010

Although photographs of bats with fungal growth similar to *G. destructans* were published in Germany in the 1980′s [13], and also taken in the 1990′s in the Czech Republic [11], there have been no confirmed records of *G. destructans* in Europe prior to 2008 [6], [12]. In 2010, *G. destructans* has been confirmed by morphological and genetic analyses from samples collected during the winters 2007/2008, 2008/2009 and 2009/2010 in six European countries [6], [11], [12]. In France, Hungary, Switzerland and Slovakia, the fungus has been confirmed from 1–2 location(s) per country, whereas it has been confirmed at 8 sites in Germany and 23 sites in the Czech Republic [6], [11], [12]. All confirmed detections of *G. destructans* in Europe have been made by isolating and/or genetically identifying the fungus from hairs, swabs or touch imprints from bats [6], [11], [12]. In Europe, eight species of *Myotis* have been observed being colonised by *G. destructans*: *M. myotis*, *M. blythii* (referred to as *M. oxygnathus* in [12]), *M. mystacinus*, *M. daubentonii*, *M. dasycneme*, *M. nattereri*, *M. bechsteinii* and *M. brandtii*. Species from other bat families were present in the caves with infected individuals (e.g. Miniopteridae: *Miniopterus schreibersii*; Rhinolophidae: *Rhinolophus hipposideros* and *R. ferrumequinum*), but no *G. destructans* has been confirmed from these species. Previous extensive surveys of cave fungi in Europe (i.e. [14], [15], [16]) or fungi associated with insects hibernating in underground sites [17] never reported *G. destructans* in their inventory, although some other species of *Geomyces* were recovered [14], [15], [16].

### New data on *G. destructans* in Europe 2003–2010

**Figure 1:**
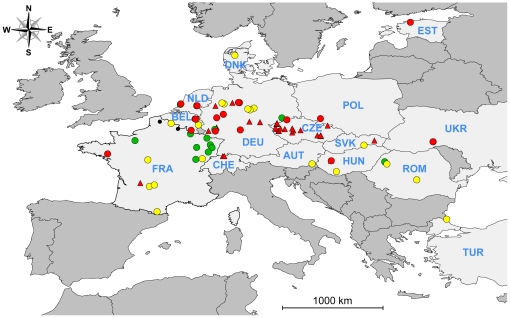
Distribution of confirmed and suspected records of *G. destructans* on hibernating bats in Europe. Data are presented for genetically confirmed records of *G. destructans* in red (circles, this study; triangles, published records), photographic evidence in yellow, visual reports in green. Dead bats from Northern France which culture and genetic analysis did not reveal the presence of *G. destructans* are depicted as black dots. Countries abbreviated names are as follows: AUT: Austria, BEL: Belgium, CHE: Switzerland, CZE: Czech Republic, DEU: Germany, DNK: Denmark, EST: Estonia, FRA: France, HUN: Hungary, NLD: Netherlands, POL: Poland, ROM: Romania, SVK: Slovakia, TUR: Turkey, UKR: Ukraine.

**Figure 2:**
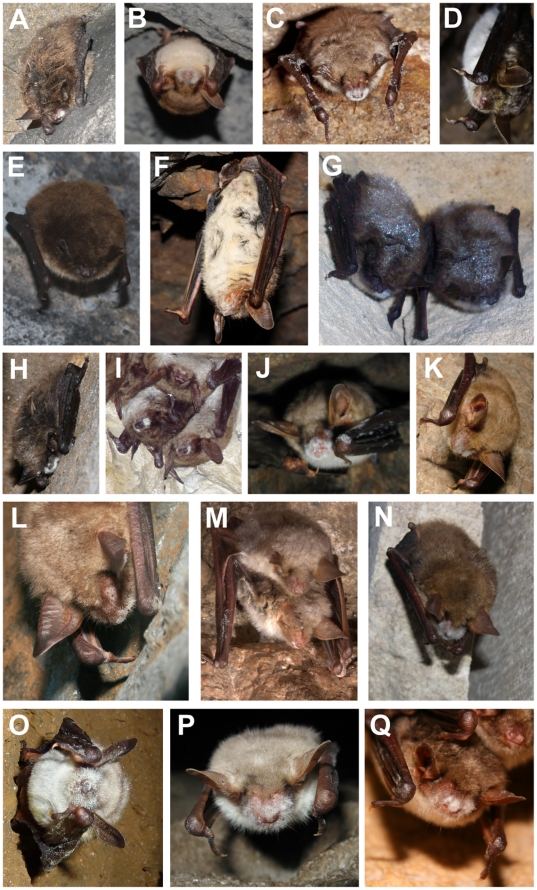
Photographic evidence showing bats with confirmed or suspected growth of *G. destructans*. Photographs of cases confirmed by genetic analysis, from (A) Estonia (*M. brandtii*, May 23^rd^ 2010, © L. Lutsar), (B) Poland (*M. myotis*, March 7^th^ 2010, © A. Wojtaszewski), (C) Germany (*M. myotis*, March 10^th^ 2010, © C. Jungmann), (D) France (*M. myotis*, March 4^th^ 2010, © Y. Le Bris), (E) Netherlands (*M. daubentonii*, March 9^th^ 2010, © T. Bosch), (F) Germany (*M. myotis*, March 23^rd^ 2010, © K. Passior) (G) Belgium (*M. mystacinus*, March 18^th^ 2010, © B. Mulkens), (H) Germany (*M. mystacinus*, March 23^rd^ 2010, © K. Passior) or bats with white-fungal growth suspected as *G. destructans* from (I) Denmark (*M. dasycneme*, March 14^th^ 2010, © B. Ohlendorf), (J) Austria (*M. myotis*, February 2^nd^ 2007, © O. Gebhardt), (K) Hungary (*M. myotis*, February 19^th^ 2010, © T. Görföl), (L) Belgium (*M. myotis*, March 7^th^ 2010, © F. Forget), (M) France (*M. myotis*, February 13^th^ 2010, © J. Vittier), (N) Ukraine (*M. myotis*, February 13^th^ 2010, © A.-T. Bashta), (O) France (*M. escalerai*/sp. *A*, June 25^th^ 2010, © F. Blanc), (P) Turkey (*M. myotis*/*blythii*, March 22^nd^ 2009, © M. Doker), and (Q) Romania (*M. blythii*, March 29^th^ 2008, © B. Szilárd).

During winter hibernation counts, a total of 107 bats from 56 sites in twelve European countries were reported to have visible white fungal growth (Tables 1, 2, 3 and Figure 1). This represents the first records from nine countries (Austria, Belgium, Denmark, Estonia, The Netherlands, Poland, Romania, Turkey and Ukraine). One hundred and five bats were alive and two of them were found dead in hibernacula. These 107 bats belonged to eight different species of *Myotis*: *M. myotis* (59), *M. dasycneme* (26), *M. mystacinus* (9), *M. daubentonii* (4), *M. myotis/blythii* (3), *M. blythii* (3), *M. nattereri* (1), *M. escalerai*/sp. *A* (1) and *M. brandtii* (1). Of these, molecular and morphological identifications of the colonising fungus were carried out in 23 cases (Table 1), while only photographic evidence was obtained for a further 50 cases (Table 2 and Figure 2). The remaining 34 cases were based on reports of visual observations of a white fungal growth on bat snouts and/or ears (Table 3), which was very similar to pictures presented in Figure 2. All 84 bats reported in Tables 2 and 3 are considered as Gd-suspects (bats showing fungal growth that is thought to be *G. destructans*).

**Table 1:** Confirmed records of *Geomyces destructans* on hibernating bats in Europe and details of the culture and genetic analyses.

Country	Lat	Lon	Date	Host species	Culture	PCR	GenBank No.
France*	49.9	4.1	04/03/2010	*Myotis mystacinus*	GuH-04032010	-‡	n/a
France*	50.6	2.5	22/02/2010	*Myotis nattereri*	ThC-22022010	-‡	n/a
France †	47.7	-2.1	04/03/2010	*Myotis myotis*	Mmyo-FR-1	+	JF502415
Belgium	49.8	5.3	03/04/2010	*Myotis myotis*	Mmyo-BE-1	+	JF502414
Belgium †	50.8	5.6	18/03/2010	*Myotis mystacinus*	Mmys-BE-1	+	JF502407
Belgium †	50.8	5.6	18/03/2010	*Myotis mystacinus*	n/a	+	n/a
Netherlands †	52.0	5.8	09/03/2010	*Myotis daubentonii*	Mdau-NL-1	+	JF502411
Netherlands	52.1	4.3	27/02/2010	*Myotis dasycneme*	Mdas-NL-1	+	JF502410
Germany †	49.7	7.4	10/03/2010	*Myotis myotis*	Mmyo-DE-12	+	JF502401
Germany	49.8	9.6	22/03/2010	*Myotis myotis*	Mmyo-DE-14	+	JF502403
Germany	50.7	13.7	20/03/2010	*Myotis myotis*	Mmyo-DE-13	+	JF502402
Germany	50.9	7.5	18/04/2009	*Myotis myotis*	Mmyo-DE-10	+	JF502399
Germany	51.2	8.1	21/03/2010	*Myotis mystacinus*	Mmys-DE-2	+	JF502409
Germany	51.2	8.1	21/03/2010	*Myotis mystacinus*	Mmys-DE-3	+	n/a
Germany	51.2	8.1	07/03/2010	*Myotis myotis*	Mmyo-DE-11	+	JF502400
Germany	51.2	8.1	07/03/2010	*Myotis myotis*	Mmyo-DE-16	+	n/a
Germany †	52.3	9.5	23/03/2010	*Myotis myotis*	Mmyo-DE-15	+	JF502404
Germany †	52.3	9.4	23/03/2010	*Myotis mystacinus*	Mmys-DE-1	+	JF502408
Hungary	47.1	17.6	24/03/2010	*Myotis myotis*	Mmyo-HU-2	+	JF502405
Hungary	47.1	17.6	24/03/2010	*Myotis myotis*	Mmyo-HU-3	+	n/a
Poland	50.8	16.7	07/03/2010	*Myotis myotis*	Mmyo-PL-1	+	JF502413
Estonia#, †	59.3	24.6	01/06/2010	*Myotis brandtii*	EsT-01062010	+	JF502412
Ukraine	48.7	26.6	17/03/2010	*Myotis myotis*	Mmyo-UA-1	+	JF502406

*Dead bat.

# Environmental sample (individual observed 23/05/2010; see text for further explanations).

† Photograph of the bat shown in Figure 2.

‡ Samples were negative for *G. destructans* but amplified another fungus.

**Table 2:** Suspected photographic records of *Geomyces destructans* on hibernating bats in Europe.

Country	Lat.	Lon.	Date	Host species	No. Individual
France	44.8	1.6	25/04/2008	*Myotis myotis*	1
France †	42.6	2.2	26/06/2010	*Myotis escalerai/sp.A*	1
France	47.7	−2.1	04/03/2010	*Myotis myotis*	1
France †	45.0	2.0	13/02/2010	*Myotis myotis*	2
France	47.3	6.2	04/03/2010	*Myotis myotis*	3
France	50.4	3.5	01/03/2008	*Myotis mystacinus*	1
France	47.2	1.4	24/02/2010	*Myotis myotis*	2
Belgium	50.8	5.6	09/02/2008	*Myotis dasycneme*	1
Belgium	50.8	5.6	20/03/2008	*Myotis daubentonii*	1
Belgium	50.8	5.6	17/01/2010	*Myotis dasycneme*	1
Belgium †	50.3	5.9	07/03/2010	*Myotis myotis*	1
Belgium	50.8	5.7	13/03/2010	*Myotis dasycneme*	1
Netherlands	52.1	4.3	26/03/2008	*Myotis dasycneme*	1
Netherlands	52.1	4.3	18/02/2008	*Myotis dasycneme*	1
Netherlands	52.0	5.7	04/03/2010	*Myotis mystacinus*	1
Denmark †	56.4	9.1	14/03/2010	*Myotis dasycneme*	2
Germany	51.8	10.8	02/02/2008	*Myotis myotis*	1
Germany	51.6	10.5	07/02/2010	*Myotis myotis*	1
Germany	51.7	10.3	20/03/2010	*Myotis myotis*	1
Germany †	52.3	9.5	23/03/2010	*Myotis mystacinus*	1
Germany	52.3	9.5	23/03/2010	*Myotis dasycneme*	1
Germany	52.1	8.2	21/03/2007	*Myotis daubenonii*	1
Germany	52.1	8.2	14/03/2007	*Myotis dasycneme*	1
Germany	52.2	8.0	04/02/2008	*Myotis myotis*	1
Austria †	46.8	16.0	07/02/2007	*Myotis myotis*	1
Hungary	47.1	17.6	24/02/2007	*Myotis myotis*	1
Hungary	47.1	17.6	23/02/2009	*Myotis myotis*	1
Hungary	46.2	18.1	03/03/2009	*Myotis myotis/blythii*	1
Hungary	48.5	20.5	18/02/2010	*Myotis blythii*	1
Hungary	47.1	17.6	19/02/2010	*Myotis blythii*	1
Hungary †	47.1	17.6	19/02/2010	*Myotis myotis*	2
Poland †	50.8	16.7	07/03/2010	*Myotis myotis*	1
Ukraine †	48.8	26.6	13/02/2010	*Myotis myotis*	1
Ukraine	48.8	26.6	17/03/2010	*Myotis myotis*	8
Romania †	46.8	22.6	29/03/2008	*Myotis blythii*	1
Romania	45.4	25.2	14/03/2009	*Myotis myotis/blythii*	1
Turkey †	41.9	27.9	22/03/2009	*Myotis myotis/blythii*	1

† Photograph of the bat shown in Figure 2.

**Table 3:** Suspected visual records of *Geomyces destructans* on hibernating bats in Europe.

Country	Lat.	Lon.	Date	Host species	No. Individual
France	49.1	6.6	06/04/2009	*Myotis myotis*	1
France	48.5	6.9	28/02/2009	*Myotis myotis*	1
France	48.3	7.1	29/03/2009	*Myotis myotis*	1
France	48.3	5.7	16/03/2008	*Myotis myotis*	1
France	47.9	6.8	03/03/2010	*Myotis myotis*	2
France	49.5	5.2	04/03/2010	*Myotis myotis*	1
France	48.9	0.3	06/02/2010	*Myotis myotis*	1
France	47.2	5.7	20/02/2010	*Myotis myotis*	3
Netherlands	52.1	4.3	10/03/2005	*Myotis dasycneme*	2
Netherlands	52.1	4.3	24/06/2006	*Myotis dasycneme*	1
Netherlands	52.1	4.3	07/03/2007	*Myotis dasycneme*	1
Netherlands	52.1	4.3	15/03/2008	*Myotis dasycneme*	3
Netherlands	52.1	4.3	30/03/2008	*Myotis dasycneme*	2
Netherlands	52.1	4.3	05/04/2008	*Myotis dasycneme*	1
Netherlands	52.1	4.3	12/04/2008	*Myotis dasycneme*	1
Netherlands	52.1	4.3	13/02/2004	*Myotis dasycneme*	2
Netherlands	52.1	4.3	05/04/2003	*Myotis dasycneme*	1
Netherlands	52.1	4.3	26/03/2008	*Myotis dasycneme*	1
Netherlands	52.1	4.3	10/03/2005	*Myotis dasycneme*	1
Germany	50.9	13.3	23/03/2010	*Myotis daubentonii*	1
Germany	49.9	7.4	14/03/2010	*Myotis myotis*	1
Ukraine	48.8	26.6	17/03/2010	*Myotis myotis*	4
Romania	47.0	22.4	08/04/2008	*Myotis myotis*	1

### *Geomyces destructans* identification

Out of a total of 107 bats with fungal growth, 22 were sampled, 16 with touch imprints and 6 with cotton swabs. The 22 bats sampled (20 alive and 2 dead) belonged to the species of *Myotis* from which *G. destructans* had been previously isolated (see list above). In some cases, we were not able to discriminate between *M. myotis* and *M. blythii* (referred to as *M. myotis/blythii*) as well as between the newly recognised *M. escalerai* [18], [19] and *Myotis* sp. *A* [20], a yet undescribed cryptic species from the *M. nattereri* species complex [18], [21]. Additionally, swab samples were collected from the tunnel wall of an Estonian hibernaculum. On the 23^rd^ May 2010, a *M. brandtii* was observed in this hibernaculum with white fungal growth on its snout (Figure 2A) but no sample was collected at the time. When the site was revisited for sample collection on the 1^st^ of June 2010, the bat had left the site so samples were collected by swabbing the wall of the tunnel where the bat was seen nine days before. Four cotton swabs were used to sample different areas a few centimetres around the location where the bat was observed. The four swabs were then streaked onto four Sabouraud's agar plates each and monitored regularly to physically remove any fungal growth that was not similar to *G. destructans*. Although the amount of fungi varied per swab sample, *G. destructans* was recovered from all four swabs, henceforth considered as a single sample, bringing the total of samples analysed to 23. No mass mortality was reported at any of the sites investigated.

Out of 23 samples investigated in the laboratory, 14 of the 16 touch imprint samples presented characteristic conidia when observed under a microscope and two of them were doubtful; none of the cotton swabs were inspected under a microscope prior to culture. Cultures from 22 of these samples were successful. The two dead bats investigated did not reveal the presence of *G. destructans* but other fungal species such as *Mucor* sp. and *Cladosporium* sp. were identified (data not shown).

DNA was isolated from the 22 cultures of which 20 showed morphological similarity to *G. destructans* (e.g. curved conidia) and from one touch imprint with unsuccessful culture attempts. Amplification and sequencing of the internal transcribed spacer (ITS) region (ITS1, 5.8S, and ITS2) was preferred over the small subunit (SSU) rDNA as it was shown to be more informative and was comparable to both European [6], [12] and North American *G. destructans* [7], [22]. All sequences obtained were identical and showed 100% similarity with previously published ITS sequences of *G. destructans* available on GenBank (retrieved on October 13^th^) [6], [7], [12], [22].

### Seasonal distribution of *G. destructans*

**Figure 3:**
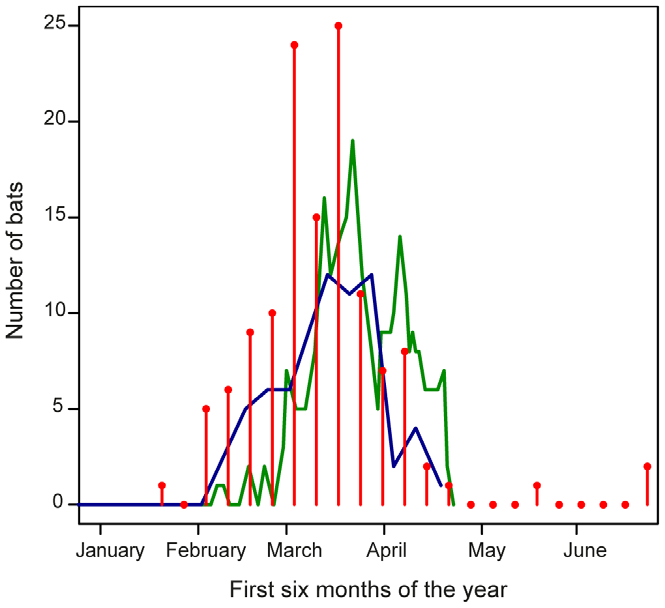
Seasonal changes of the number of live bats reported with white fungal growth in Europe. The number of bats with visible white fungal growth at an hibernaculum in Germany was monitored during the winter 2006/2007 (blue line) and the winter 2007/2008 (green line). The vertical red lines represent the number of Gd-suspect bats (or confirmed) observed across twelve European countries (n = 127) from 2003 until 2010. In the X-axis, the thick marks represent the start of each month.

**Figure 4:**
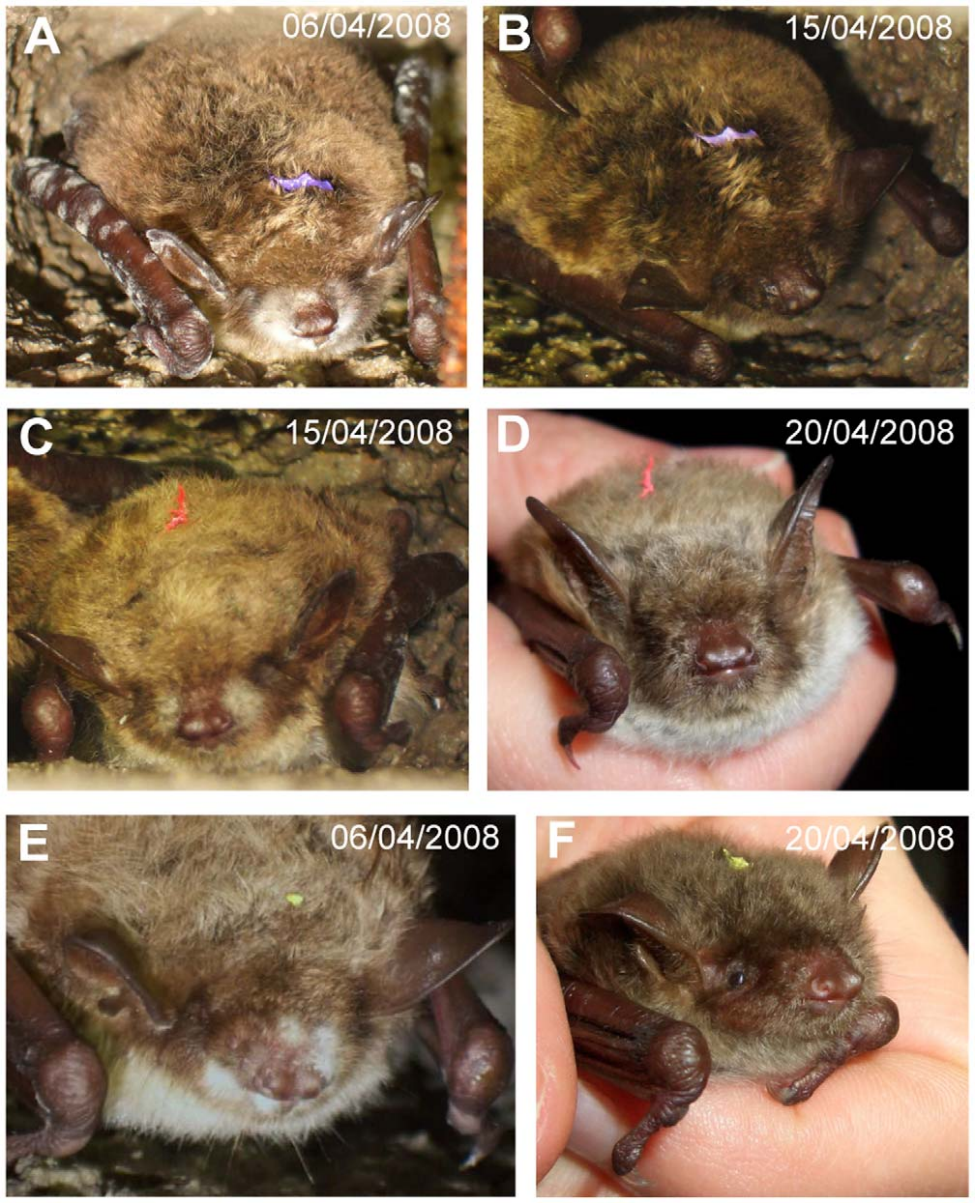
Indirect evidence of bats grooming off *G. destructans* during hibernation. Photographic evidence showing three different *M. dasycneme* individuals (A–B, C–D and E–F) observed at two different dates, first with visible fungal growth (A, C, E) and later without visible fungal growth (B, D, F). The bat in A–B changed its position within the hibernaculum whereas the other two (C–D and E–F) were captured when leaving the hibernaculum (© V. Korn).

The monitoring of one site over two consecutive winters showed an absence of Gd-suspect bats from September until the end of January (Figure S1). The first Gd-suspect bats were reported in February each year (16/02/2007 and 07/02/2008) and their numbers peaked in March (Figure 3). In April, the total number of bats and the number of Gd-suspect bats decreased as bats left the hibernacula. However, as the number of Gd-suspect bats decreased more slowly than the total numbers of bats, the highest prevalence was observed in April (Figure S1). Prevalence varied between years for the same period of the year and reached values in the range of 18–25% in 2007 (14^th^–28^th^ March) or 28–55% in 2008 (13^th^–28^th^ March) when the numbers of Gd-suspect bats are at the highest. The distributions of reported cases were similar between the two years, although more cases were reported in April in the winter 2007/2008 (Figure 3). In April 2008, the monitoring of three marked bats with white fungal growth clearly showed that after a bat had changed its position within the hibernaculum or when it was leaving the hibernaculum, the visible white fungal growth disappeared (Figure 4), most likely as a result of self-grooming.

The temporal distribution of reported cases of live Gd-suspect bats from throughout Europe (this study, n = 105) was combined with information available from previously reported cases of *G. destructans* [6], [12] (n = 22) to investigate the seasonal variation across multiple sites in Europe. The temporal range of reported cases of Gd-suspect live bats and bats confirmed with *G. destructans* (n = 127) was not evenly distributed throughout the winter/spring, with about 2/3^rd^ of the cases reported in March (81/127; Figure 3). The number of reported cases more than doubled between February (30 cases) and March (81 cases). The earliest case was reported on January 17^th^ from Belgium and the three latest cases were observed on May 23^rd^ in Estonia, in June 24^th^ in the Netherlands and June 25^th^ in France (Tables 1, 2, 3, Figures 2A and 2O).

## Discussion

### Presence of *G. destructans* in Europe

*G. destructans* was first identified in Europe in 2008–2009 [6], [12] but increasing photographic evidence suggest that the fungus was present in Europe well before this date (this study, [11], [13]). Most previous studies investigating fungi in European caves, including bat guano [14], [15], [23], [24] reported *Geomyces* species, but none had curved conidia so far typical of *G. destructans*. In the Czech Republic, Kubátová & Dvořák [17] investigated fungi associated with insects hibernating in underground sites but did not find *Geomyces* species. To our knowledge, only one study in Europe has investigated fungi present in bats' skin and hair samples where, based on our current knowledge, *G. destructans* is most likely to be found. During the winter 1999/2001, Larcher et al. [25] collected 25 samples of hair and skin swabs from six species, including three *Myotis myotis*, but did not find any *Geomyces* species. It is important to note that most fungal cultures have been carried out at temperatures above 24–25°C, temperatures at which *G. destructans* does not grow [4], [22], which could explain why although present, this fungal species had never been reported in Europe before the study of Puechmaille et al. [6].

Combining previously published data from France, Germany, Switzerland, Hungary, The Czech Republic and Slovakia [6], [11], [12], additional data collected from France, Germany and Hungary (this study), and new data from Belgium, The Netherlands, Poland, Estonia and Ukraine (this study), we demonstrate here that *G. destructans* is widespread in Europe. We consider the photographic evidence of bats with white fungus matching the characteristic growth pattern (e.g. Figure 2; pictures from Romania and Turkey) to most likely represent *G. destructans*, because so far all tested live European bats with such white fungal growth on their nose, similar to Figure 2, have been confirmed to carry that species of fungus. These findings further support the fact that *G. destructans* is widespread across Europe. However, to confirm the presence of *G. destructans* in Europe prior to 2008, historical collections of bat specimens (or cave soil samples), especially specimens collected during the hibernation period, should be screened for the fungus.

As depicted in Figure 1, most cases of bats with *G. destructans* (confirmed and suspected) have been found from North-eastern France through Belgium, The Netherlands, Germany and the Czech Republic. However, it is not clear whether this pattern reflects an actual higher occurrence and/or prevalence of the fungus in these regions or if it is at least partly due to sampling bias, whereby the fungus is more likely to be detected in regions with a higher number of underground sites visited every winter or in regions were the fungus is specifically sought. In our opinion, it is most likely that this large-scale pattern is due to a sampling bias. For example, the largest number of sites with *G. destructans* in any European country was reported from the Czech Republic (76 localities with suspected or confirmed *G. destructans*) were most sites have been searched for signs of the fungus (>800 hibernacula) [11].

### *G. destructans* growth on bats

The clear seasonal peak in the number of observations of bats with white fungal growth indicates an increasing prevalence or detectability of *G. destructans* as winter passes. This suggests that bats might acquire *G. destructans* late during the hibernation period or that the fungus is carried by bats at the onset of hibernation but needs time to develop the visible white fungal growth due to the phenology of the fungus. Therefore, the absence of visible white fungal growth on bats when observed with the naked eye may not directly reflect the absence of *G. destructans*, but rather just the absence of visible fungal colonies. Further complicating matters, our ability to detect *G. destructans* growth on bats can substantially differ with proximity to the bats (i.e. low ceiling *versus* high ceiling) or the location of the bat (ceiling *versus* crevices).

Our results confirm the suggestion of Martínková et al. [11] by showing that during the hibernation period, bats can remove the fungus from their snout, ears and wings to a point where the fungus is no longer visible to the naked eye, although some spores might still be present on their skin. During hibernation, bats arouse every two weeks on average [26], [27] and if bats consistently groom off the fungus on these occasions, our ability to visually detect the fungus, if present, will be considerably reduced. We also showed that towards the end of the hibernation period, bats were emerging from the hibernaculum without visible signs of the fungus despite showing visible white fungal growth from two weeks to five days before leaving the hibernaculum. It would be important to investigate whether bats carry spores out of hibernacula and as a result could possibly contaminate maternity roosts and maternity mates as suggested by Hallam and McCracken [28].

### Factors affecting *G. destructans* prevalence

Although it is not possible to clearly identify the mechanism responsible for the sudden increase in the prevalence of *G. destructans* in late February and March, these data suggest that shorter winter periods should be associated with lower prevalence. This prediction seems to hold as in the Mediterranean region, where hibernation periods are shorter [29], no bats with visually conspicuous fungal growth have yet been reported during winter cave surveys. The case reported from Southern France (June 25^th^ 2010, Figure 2O) was found in the Pyrenees mountains at *ca.* 1700 m a.s.l. and hence, is not considered typical of the Mediterranean climate. It is nevertheless too early to conclude on this association between *G. destructans* prevalence and the hibernation duration, as other factors would need to be considered such as for example, the higher temperature observed in hibernacula in the Mediterranean region compared to other regions in Europe [29]. Higher temperatures in hibernacula have been associated with more frequent arousals in *Rhinolophus ferrumequinum* [30], [31], [32]. Considering that this association holds for other species, as a consequence of more frequent arousals, bats are expected to groom more often and therefore, reduce the probability of a visible fungal growth to develop. More surveys and strategic sampling efforts are needed to uncover whether the length of the hibernation period and/or climatic conditions have a direct or indirect effect on the growth rates, prevalence, and detectability of *G. destructans* on bats.

It is crucial that the change in prevalence or detectability over the hibernation period is considered when comparing prevalence across sites and/or years. Our results from monitoring one site throughout the hibernation period over two consecutive years as well as reported cases from multiple sites in multiple years show that bats with fungal growth are first seen in January, the number of cases slowly increases into February and peaks in March, then in April when bats emerge from hibernation it drops again. Our results are in agreement with recent results from the Czech Republic where in the winter 2009/2010, the number of sites with bats with white fungal growth increased from 4.1% in January/February (33/800 sites; regular bat monitoring) to 77.5% in late February/March (76/98 sites; additional inspections) [11]. The Czech study reported that this increase in *G. destructans* prevalence was “*suggestive of an epizootic spread of the fungus*” [11]; we propose an alternative explanation whereby the increase in prevalence of *G. destructans* in late winter (March) might regularly (yearly) occur in Europe but has gone unnoticed. Nearly all hibernation counts in previous years were carried out between December and mid-February when prevalence/detectability of *G. destructans* is low, but not in March [33] when the prevalence/detectability of *G. destructans* is at its highest (Figure 3). Although the total numbers of bats in the hibernacula decreased through April as bats left for the maternity colonies, our results show that there is a high probability of fungal growth developing on the remaining individuals. This further supports our hypothesis proposed above and links the duration of the hibernation period with the prevalence of *G. destructans*. By increasing the sample size, some cases might be reported earlier in the hibernation season or later through the summer, but we expect that the general pattern observed will not change. Despite these difficulties in assessing the occurrence of the fungus on bats, our data are consistent with other studies [6], [11], [12], and also demonstrate that the most commonly encountered bat species with *G. destructans* in Europe is the largest species of *Myotis* on the continent, *Myotis myotis*. In countries/regions (i.e. the Netherlands, Northwest Germany) where *M. dasycneme* is more commonly encountered in hibernacula, *G. destructans* prevalence can reach high levels in that species. It is interesting to note that neither *Pipistrellus pipistrellus* nor *Miniopterus schreibersii* have been observed with *G. destructans* [6], [11], [12], although these two species are known to hibernate in aggregations of tens of thousands of individuals, especially the latter [34], [35], [36], [37]. Although rare, hibernacula of a few thousands and up to about 34,000 individuals are also known for species of *Myotis* in Europe [34], [35], [38], [39], [40], [41].

### *G. destructans* outside of the hibernation period

We observed three individual bats with white fungal growth around their nose (one confirmed as *G. destructans*) from May and June, when they were still torpid in cold underground sites. This represents the first mention of individuals with *G. destructans* colonisation outside of the hibernation period and raises questions about the role of these individuals in the persistence of the fungus in bat populations. During the summer period, while females aggregate in colonies to raise their young, it remains largely unknown where males are roosting (e.g. [42]). Furthermore, during the swarming season in late summer/autumn, large numbers of individuals aggregate in caves, mines or tunnels and come in close contact with each other (chasing, mating) [42], [43], [44], [45], [46], [47], which could represent an opportunity for *G. destructans* to be transmitted between individuals.

We isolated *G. destructans* from the environment surrounding hibernating bats. The presence of viable spores of *G. destructans* on the surfaces of hibernation sites has huge implications for the understanding of disease transmission mechanisms and disease modelling [28]. It seems likely that cave walls could serve as a passive vector and/or reservoir for *G. destructans* spores. It is not yet known how long these spores can remain viable but fungal spores generally remain viable for extended periods. Bats entering these sites in autumn (for swarming and/or hibernation) could become contaminated with spores of *G. destructans* left from bats infected during the previous winter. In North-America, Lindner et al. [48] successfully amplified ITS sequences identical to *G. destructans* DNA from soil samples collected during the winter 2008–2009 at three bat hibernacula and stressed the importance of considering the environment as a reservoir for *G. destructans* and in the dynamics of WNS transmission. Our results confirm this and further suggest that more work is needed to understand the persistence of *G. destructans* on hibernacula walls (reservoir or passive vector) where they are in physical contact with bats.

### Insights into the origin of *G. destructans* and WNS

The wide distribution of *G. destructans* in Europe and the absence of associated mortality supports the hypothesis that *G. destructans* has co-evolved with European bats and only recently arrived in North America where it is causing unprecedented mass mortalities [6], [7], [11], [12]. Alternatively, *G. destructans* could have been present on both continents and a virulent strain could have evolved in North-America. Until the relationships between *G. destructans* populations across continents are clarified, precautions should be taken to minimise the chances of transcontinental movement of viable *G. destructans* [49].

During the two years monitoring at one site in Germany where *G. destructans* prevalence reached high levels in March-April, not a single dead bat was found. This is in agreement with previous studies [6], [11], [12] reporting that the presence of *G. destructans* in bats from Europe is not associated with mass mortality. This sharply contrasts with mass mortalities reported in North America where hundreds or thousands of dead bats are found in hibernacula towards the end of the hibernation period. Recent pathological investigations of bats dying from WNS in North America led Cryan et al. [50] to propose that mortality was caused by important disruptions of wing-dependant physiological functions due to infection by *G. destructans*. In North America, the fungus deeply invades wings tissues [2] and causes damages that are thought to alter homeostasis and water balance, resulting in more frequent arousals than bats can afford with their fat reserves, leading to death by starvation [50]. The pathology associated with *G. destructans* colonisation in Europe is not yet known. We believe that the first step in understanding mortality differences between bats from Europe and North America rely on understanding pathological differences incurred by the fungus on the bats' wings. As a result, we urge the necessity to carry out pathological investigation of live bats from Europe colonised by *G. destructans*. Despite the absence of mortality associated with the presence of *G. destructans* in Europe, it would be necessary to investigate whether chronic infections with the fungus are compromising the health of individuals, especially in *M. myotis* and *M. dasycneme*, which show high prevalence of the fungus towards the end of the hibernation period.

Phylogeographic studies of European bat species have shown that in the last 100,000 years, some species colonised Europe from Western Asia [51], including *Myotis blythii* [52], [53] which has been found with *G. destructans* [12]. Assuming that *G. destructans* can be transported over long distances by bats, we speculate that the distribution of *G. destructans* is probably not limited to Europe and possibly extends eastwards into Russia, Western and Central Asia. Further surveys are necessary to clarify the global distribution of *G. destructans*.

### Conclusions

We have shown here that *G. destructans*, the most likely causative agent of WNS in North America, is widespread in Europe, but is not associated with mass mortality. The prevalence of visible fungal growth on bats increases in February/March before sharply decreasing when bats emerge from hibernation. We also isolated viable *G. destructans* from the walls of an underground site suggesting that the hibernacula could act as passive vectors and/or reservoirs for *G. destructans* and therefore, might play an important role in the transmission process. Further research is needed to clarify the global prevalence of *G. destructans* and identify variables (e.g. temperature, humidity and hibernation length) explaining regional differences. Finally, further research is needed in different parts of the globe, especially temperate region of the Northern and Southern hemispheres, to precisely determine the global distribution of *G. destructans*.

## Materials and Methods

### Sample collection

During ongoing population censuses carried out at hibernacula in different countries across Europe and during additional hibernacula surveys carried out for the purpose of this study, information on bats with visible white fungal growth on snouts and/or ears was recorded. Whenever possible, sterile dry cotton swabs [6] or adhesive tape touch imprints [12] were used to collect fungal material from the bats. In Estonia, samples were collected from the wall of the tunnel where a bat with characteristic white fungus was observed nine days prior to the sampling. Where no sample collection was possible, a photograph was taken of the bat (photographic record). In cases where neither sample collection nor photographic evidence was obtained, the record was classified as visual observation. Live hibernating bats with powdery, white fungal growth on their noses were considered suspects of infection by *G. destructans* (Gd-suspects) but not suspected of having WNS. There is presently no data supporting the occurrence of WNS in Europe and the co-occurrence of the fungus with lesions characteristic of WNS [2] has not (yet) been reported in Europe [12], [54]. Although, prevalence of *G. destructans* can reach high levels in some European species (i.e. *Myotis myotis*, *M. dasycneme*) in late winter (especially in March), it can be expected that by chance alone some bats dying from causes unrelated to the presence of *G. destructans* will also be carrying the fungus. Unless the criteria for the diagnosis of WNS are met (confirmation by histo-pathology and PCR) [2] WNS should not be assumed as a cause of mortality in dead bats found in hibernacula of Europe. Various species of fungi have been identified on dead bats [12], [55], most of them likely being saprophytes that colonise bat carcasses *post-mortem*.

### Fungal cultures

In the laboratory, samples were treated as in [6] for swabs and following [12] for touch imprints. Briefly, swabs were streak-plated onto plates of Sabouraud's agar, supplemented with 0.1% mycological peptone. For touch imprints, small areas with fungal conidia characteristic of *G. destructans* were identified by light microscopy and the tape was disinfected and excised before being transferred for culture to Sabouraud's agar. The plates were sealed with parafilm and incubated inverted in the dark at 10°C. A fungal growth developed within 14 days, from which single spore cultures were established.

### Molecular identification

Each culture was sequenced for one molecular marker, the rRNA gene internal transcribed spacer (ITS, *ca.* 930 bp.) region (ITS1, 5.8S, and ITS2) to further confirm species identity. The DNA extraction, PCR amplification and DNA sequencing followed protocols described in Puechmaille et al. [6]. Briefly, DNA was extracted using the Qiagen Blood and Tissue kit following the manufacturer's instructions with slight modifications (after step 3, we added an incubation time of 10 minutes at 70°C). PCR reactions were carried out in 25 µL containing 1 µL of DNA extract (at 10–75 ng/µL), 1.5 mmol/L MgCl_2_, 0.4 µmol/L each primer (Forward: ITS4, 5′-TCCTCCGCTTATTGATATGC -3′; Reverse: ITS5, 5′- GGAAGTAAAAGTCGTAACAAGG -3′; [56]), 0.2 mmol/L dNTP, 1x PCR buffer and 1 U Platinum Taq DNA Polymerase High Fidelity (Invitrogen). PCR cycling conditions were: initial step 15′ at 95°C, then 10 cycles of 30″ at 95°C, 1′45″ at 60°C (reduce of 2°C every 2 cycles), 1′ at 72°C, following by 30 cycles of 30″ at 95°C, 1′45″ at 50°C and 1′ at 72°C. PCR products were purified and sequenced by Macrogen Inc. (Seoul, Korea) in both directions using the PCR primers. Complementary sequences were assembled and edited for accuracy using CodonCode Aligner 3.0.3 (www.codoncode.com/aligner/download.htm).

### Monitoring of visible fungal growth on bats

One site situated in Northwest Germany (Latitude: 52.1; Longitude: 8.2) near the city of Osnabrück was monitored over two consecutive winters, 2006/2007 (5^th^ September until 19^th^ May) and 2007/2008 (28^th^ August until 23^rd^ April). The monitoring consisted of counting the total number of bats at the site as well as the number of bats with visible white fungal growth similar to the pictures presented in Figures 2 and 4. The counts were done by the same person (V. Korn) every 4 days on average during the first year and every 2.5 days on average during the second year. The procedures complied with guidelines of the American Society of Mammalogists and were carried out under permit number FBD7.2 60 from the Administration of the County of Osnabrück, Department of Environment.

## Supporting Information

Figure S1 Monitoring of bats at an hibernaculum in Germany during (A) the winter 2006/2007 (September 5^th^ 2006 until April 19^th^ 2007) and (B), the winter 2007/2008 (August 28^th^ 2007 until April 23^rd^ 2008). The blue line represents the total number of bats counted whereas the green line represents the number of bats with visible white fungal growth (Gd-suspects). Dotted vertical lines separate counts from each month. Note that the number of counts per month was not equal between months. In (B), the black line represents the total number of bats counted whereas the blue line represents the total number of bats bar one portion of the hibernaculum where bats grouped densely (*ca*. 20 individuals) and did not allow a reliable identification of the number of bats with white fungal growth. The green line represents the number of bats with visible white fungal growth (Gd-suspects) counted at the hibernaculum without considering individuals densely grouping at one place in the hibernaculum. The group of about 20 individuals formed while the hibernaculum was partially flooded, likely as a result of bats changing position to avoid drowning. Note that the right Y-axis scale is different between (A) and (B).
(PDF)
