# Supplementary material for: Pan-European Distribution of White-Nose Syndrome Fungus (Geomyces destructans) Not Associated with Mass Mortality
Source: PLoS One. 2011 Apr 27;6(4):e19167. doi: 10.1371/journal.pone.0019167 (PMC3083413; doi:10.1371/journal.pone.0019167)
Supplement: Figure S1 — Monitoring of bats at an hibernaculum in Germany during (A) the winter 2006/2007 (September 5th 2006 until April 19th 2007) and (B), the winter 2007/2008 (August 28th 2007 until April 23rd 2008). The blue line represents the total number of bats counted whereas the green line represents the number of bats with visible white fungal growth (Gd-suspects). Dotted vertical lines separate counts from each month. Note that the number of counts per month was not equal between months. In (B), the black line represents the total number of bats counted whereas the blue line represents the total number of bats bar one portion of the hibernaculum where bats grouped densely (ca. 20 individuals) and did not allow a reliable identification of the number of bats with white fungal growth. The green line represents the number of bats with visible white fungal growth (Gd-suspects) counted at the hibernaculum without considering individuals densely grouping at one place in the hibernaculum. The group of about 20 individuals formed while the hibernaculum was partially flooded, likely as a result of bats changing position to avoid drowning. Note that the right Y-axis scale is different between (A) and (B). (PDF) [file pone.0019167.s001.pdf]

Supporting information for: Pan-European Distribution of White-Nose Syndrome Fungus (*Geomyces destructans*) not Associated with Mass Mortality

Sébastien J. Puechmaille*, Gudrun Wibbelt, Vanessa Korn, Hubert Fuller, Frédéric Forget, Kristin Mühldorfer, Andreas Kurth, Wieslaw Bogdanowicz, Christophe Borel, Thijs Bosch, Thomas Cherezy, Mikhail Drebet, Tamás Görföl, Anne-Jifke Haarsma, Frank Herhaus, Guénael Hallart, Matthias Hammer, Christian Jungmann, Yann Le Bris, Lauri Lutsar, Matti Masing, Bart Mulkens, Karsten Passior, Martin Starrach, Andrzej Wojtaszewski, Ulrich Zöphel and Emma C. Teeling

* Corresponding Author E-mail: s.puechmaille@gmail.com

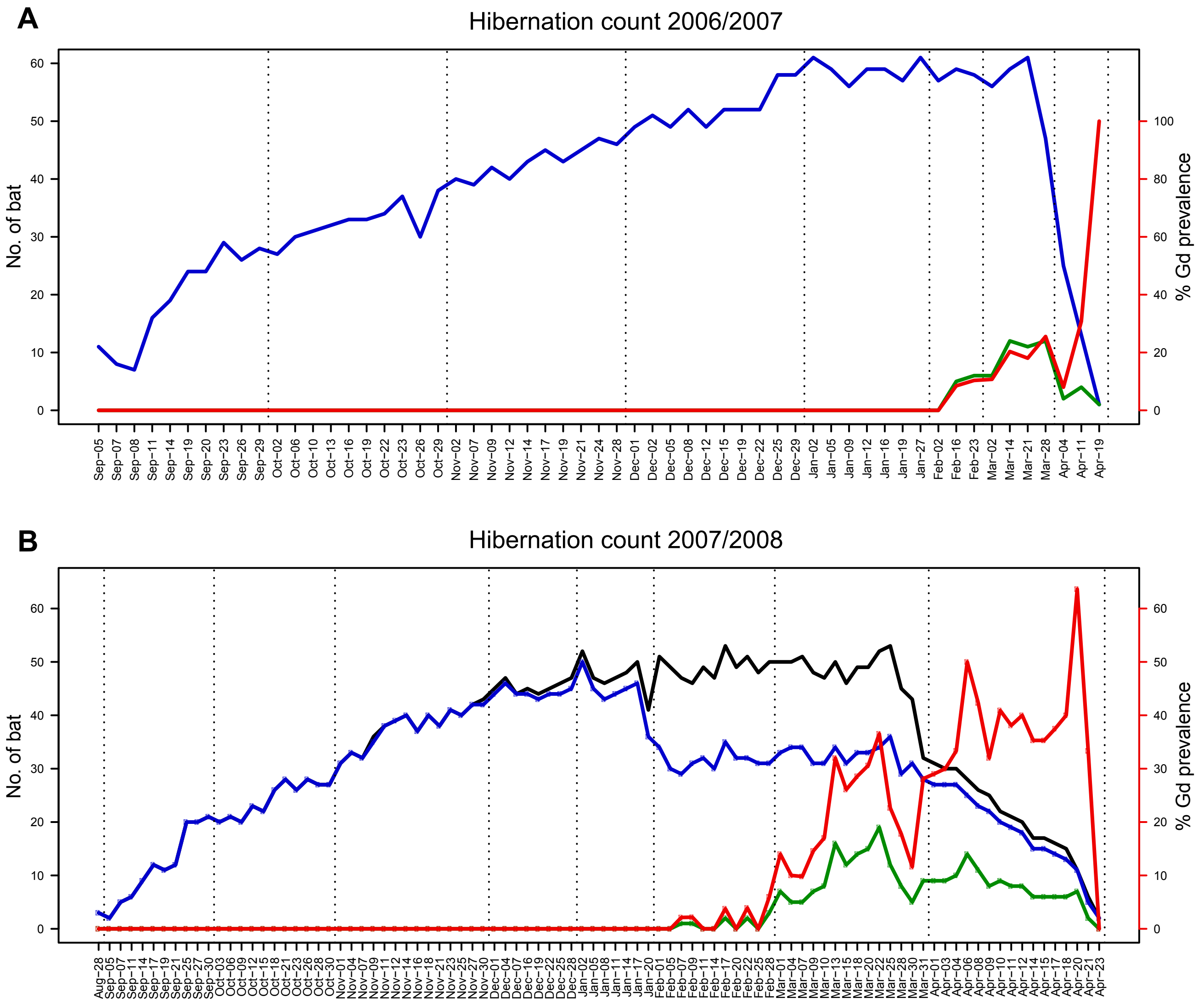

Figure S1. Monitoring of bats at an hibernaculum in Germany during (A) the winter 2006/2007 (September 5th 2006 until April 19th 2007) and (B), the winter 2007/2008 (August 28th 2007 until April 23rd 2008). The blue line represents the total number of bats counted whereas the green line represents the number of bats with visible white fungal growth (Gd-suspects). Dotted vertical lines separate counts from each month. Note that the number of counts per month was not equal between months. In (B), the black line represents the total number of bats counted whereas the blue line represents the total number of bats bar one portion of the hibernaculum where bats grouped densely (ca. 20 individuals) and did not allow a reliable identification of the number of bats with white fungal growth. The green line represents the number of bats with visible white fungal growth (Gd-suspects) counted at the hibernaculum without considering individuals densely grouping at one place in the hibernaculum. The group of about 20 individuals formed while the hibernaculum was partially flooded, likely as a result of bats changing position to avoid drowning. Note that the right Y-axis scale is different between (A) and (B).
